# Sex as a risk factor regarding presbyopia in the rhesus monkey

**DOI:** 10.1371/journal.pone.0300476

**Published:** 2024-04-18

**Authors:** Mary Ann Croft, Jared P. Mcdonald, Julie Kiland, Julie A. Mattison, George S. Roth, Don Ingram, Paul L. Kaufman

**Affiliations:** 1 Department of Ophthalmology and Visual Sciences, University of Wisconsin-Madison, Madison, WI, United States of America; 2 Translational Gerontology Branch, National Institute on Aging, NIH, Dickerson, MD, United States of America; 3 GeroScience Inc. and Prolongevity Technologies LLC, Pylesville, MD, United States of America; 4 Pennington Biomedical Research Center, Louisiana State University, Baton Rouge, LA, United States of America; 5 Wisconsin National Primate Research Center, University of Wisconsin-Madison, Madison, WI, United States of America; 6 McPherson Eye Research Institute, University of Wisconsin-Madison, Madison, WI, United States of America; University of Warmia, POLAND

## Abstract

**Purpose:**

To determine the effect of sex as a risk factor regarding presbyopia.

**Methods:**

Maximum accommodation was pharmacologically induced (40% cabachol corneal iontophoresis) in 97 rhesus monkeys (49 males and 48 females) ranging in age from 8 to 36 years old. Accommodation was measured by Hartinger coincidence refractometry.

**Results:**

Accommodative amplitude measured refractometrically decreased with age, and the rate of change was not different between males and females (p = 0.827).

**Conclusions:**

Presbyopia is essentially sex neutral, and no one is spared. There may be modest variations between different populations for various reasons, but essentially it is monotonously predictable. At present there is no biological therapeutic.

## 1. Introduction

The accommodative mechanism of the rhesus monkey and the human are virtually identical [1], and both species develop presbyopia at the same rate relative to lifespan [2, 3]. The rhesus monkey (*Macaca mulatta*) provides the best model to study human accommodation and presbyopia [1, 4–12]. and with an aging rate of approximately 2.5:1 to humans, these changes are evident much sooner. We have studied dynamic accommodation and the magnitude of the movements made by components of the accommodative apparatus in both human and monkey eyes with comparable results [1, 4–14].

In recent years there has been great interest to determine the influence of sex on the development of ocular diseases, including presbyopia. In this study we quantitatively evaluated the effect of sex as a risk factor for presbyopia in the rhesus monkey, which may have implications for human presbyopia.

## 2. Materials and methods

### 2.1. Subjects

The present study included a total of 97 (49 males and 48 females) rhesus monkeys (Macaca mulatta), representing the same cohort of monkeys described in a previous study by Mattison et al., 2005 [15]. The focus of the present analysis was on the differences between male and female monkeys. A description of the materials and methods was previously given by Mattison et al., [15] and we include it here (in italics):

*“The monkeys were part of an on-going study of aging and caloric restriction (CR) in non-human primates at the National Institute on Aging [16]. The monkeys ranged in age from 2 to 23 years at the time CR was initiated. Data were collected from male monkeys that had been maintained on 30% CR for 12 or 13 years, and females were on 30% CR for 8 years. Animal husbandry and diet composition were described in detail in a previous study [16]. Monkeys were housed individually indoors but had extensive auditory, visual, and olfactory interaction with other monkeys in the vivarium. Rooms were maintained on a 12 hr light (0600 hours):12 hr dark (1800 hours) cycle and were controlled for temperature (22–28 C°) and humidity (50% - 60%). Monkeys in both the control (CON) (n = 54) and CR (n = 43) groups were fed two meals a day (0700 and 1400 hours). The daily food allotment offered to CR monkeys was 30% less than that given to age and body weight-matched monkeys in the CON group. Diet composition did not differ between the two groups. As such, the experimental manipulation was a reduction in total caloric intake. All experiments were performed with approval of the NIA Institutional Animal Care and Use Committee and in accordance with the ARVO Statement for the Use of Animals in Ophthalmic and Vision Research.”*

### 2.2. Housing and health monitoring

As these are the same group of monkeys as Mattison et al., 2005 [15] the housing and monitoring are the same as stated by the authors in Ingram et al 1990 [16].

No animals were sacrificed for this study and all animals were returned to the home colony following these experiments. Since no invasive procedures were undertaken for this study the animals should have had minimal pain or discomfort, thus the animals received anesthesia to allow for observers to take the necessary measurements.

### 2.3. Methods

Each animal was anesthetized with an average dose of 3–5 mg/kg IM Telazol (1:1 ratio of tiletamine and zolazepam) (Fort Dodge Animal Health, Fort Dodge, IA) injected intramuscularly in the hind leg in combination with 0.1–0.2 mg/kg acepromazine maleate (Boehringer Ingelheim Vetmedica, St. Joseph, MO).

Refraction was measured using a Hartinger coincidence refractometer (aus Jena, Jena; Zeiss, Germany) before and after pharmacologically inducing accommodation. A cholinomimetic drug, 40% carbamylcholine chloride, (CARB; Sigma, St. Louis, MO), was applied by corneal iontophoresis [17], 2 X 15 seconds (CARB), to one eye, to induce maximum accommodation [18]. Carbachol induces accommodation by stimulating the ciliary muscle to contract, releasing tension on the anterior zonules, allowing the lens to change shape and position, and thereby increase its refractive power. The Hartinger refractometer objectively determines the refractive state of the eye. Following carbachol administration, the optical power shift on the instrument from baseline required to realign the images of the test pattern by a trained technician represents the accommodative amplitude.

### 2.4. Statistical analyses

The data were analyzed using linear regression techniques (via Minitab). Baseline measurements obtained from the left and right eye were not statistically different; therefore, analyses were performed using the CARB-treated eye for baseline data. Standard errors (SE) are presented immediately following estimates in the form: estimate MSE. The level of significance for all tests was p ≤ 0.05.

## 3. Results

**Fig 1:**
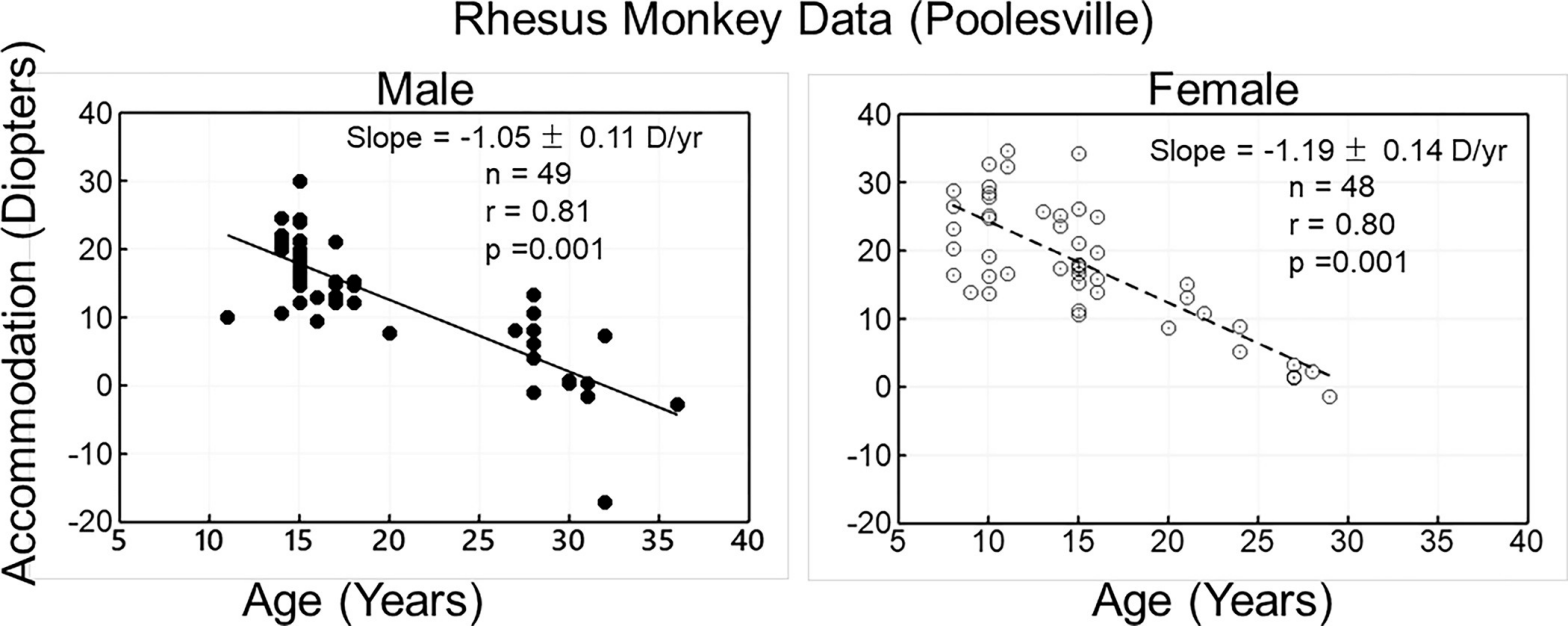
The data are Carbachol (CARB)-induced accommodative amplitude as a function of age in male (left panel) and female (right panel) rhesus monkeys. Accommodative amplitude declined with age in both the male and female monkeys. There was no difference between the gender groups (p = 0.827). Because more young females vs young males were included in the study we conducted a test for the interaction and the p value for the interaction is p = 0.430. Thus, there was not a significant interaction.

**Fig 2:**
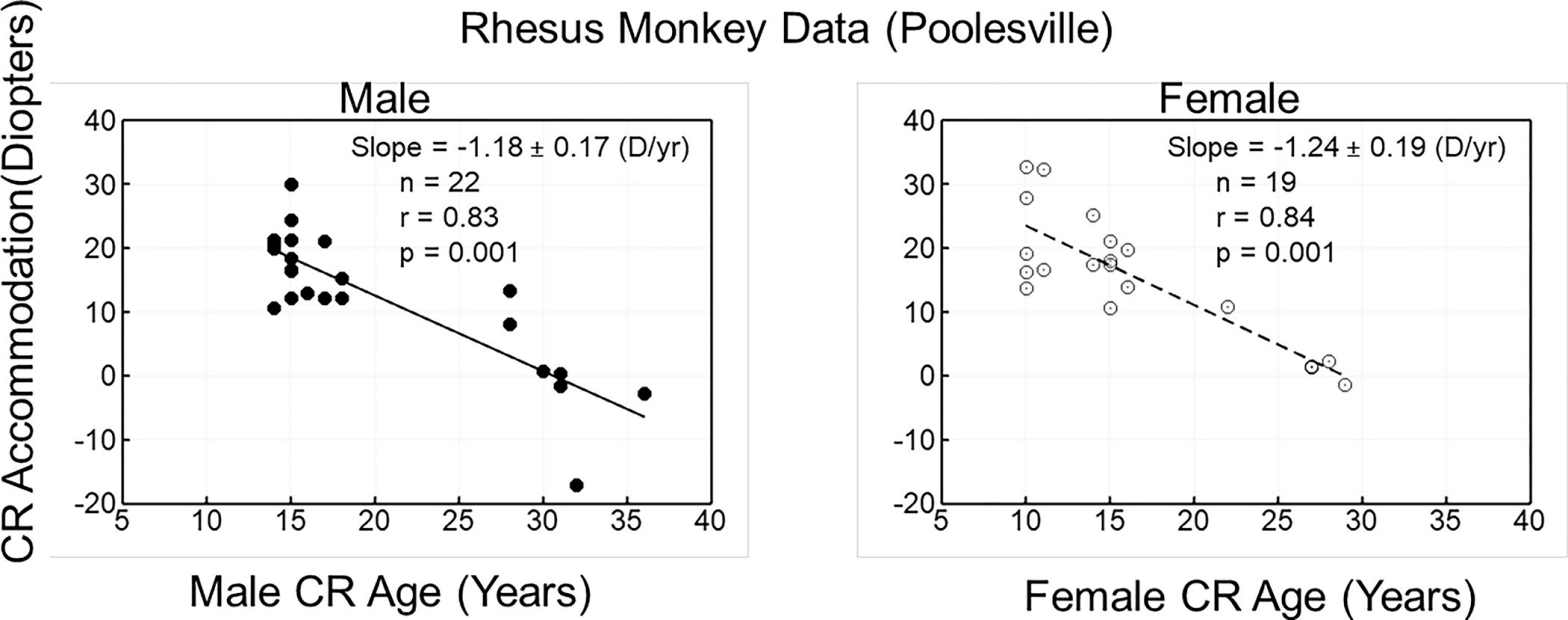
The data are Carbachol (CARB)-induced accommodative amplitude as a function of age in calorie restricted (CR) male (left panel) and CR female (right panel) rhesus monkeys. Accommodative amplitude declined with age in both the CR male and CR female monkeys. There was no difference between the gender groups.

**Fig 3:**
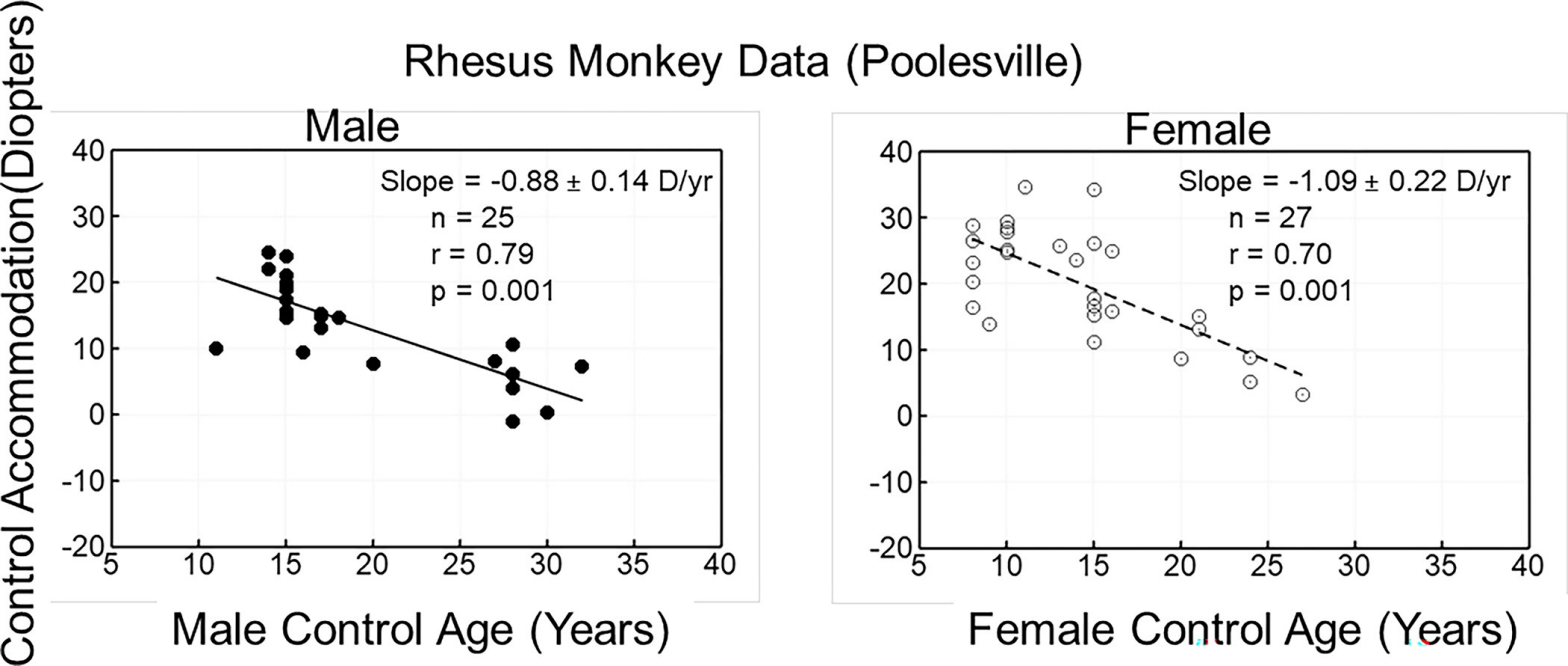
The data are Carbachol (CARB)-induced accommodative amplitude as a function of age in control male (left panel) and control female (right panel) rhesus monkeys. Accommodative amplitude declined with age in both the control male and control female monkeys. There was no difference between the control gender groups. Control Monkeys = fed ad libitum.

Accommodative amplitude measured refractometrically decreased with age, and the rate of change was not different between males and females (p = 0.827; t = -0.22) (Fig 1). There was no sex effect when separated by diet group either (CR: Fig 2) and (CON: Fig 3).

In order to account for the greater number of young females vs. young males in this study, we conducted a test for the interaction to compare the differences between males in the CR group versus females in the CON group. It was determined that the effect of CR was not sex dependent (p = 0.430) Again, the results of males and female accommodation were grouped together for studies of accommodation and presbyopia.

## 4. Discussion

The current analyses demonstrate that male and female rhesus monkeys are equally susceptible to presbyopia. This finding is consistent with what has been reported in in human subjects by Hickenbotham et al 2012 [19]. The conclusions from this meta-analysis of human subject research (which included review of nine cross-sectional studies from other scientists) the authors state: “there are no significant sex differences in presbyopia due to focusing ability when measured by accommodative amplitudes. However, there were significant differences in the add requirements for near vision spectacles for men vs. women of the same age; these differences were likely due to variations in preferred viewing distances or to uncorrected hyperopia.” [19, 20].

In conclusion, studies of accommodative amplitude and presbyopia may include either sex and the data (male and female) should be grouped together. This analysis also provides further evidence that the rhesus monkey is a suitable/reliable/useful model for studying human accommodation and presbyopia.

## Supporting information

S1 Data (XLSX)
